# Responses towards eyefluke (*Diplostomum pseudospathaceum*) in different genetic lineages of rainbow trout

**DOI:** 10.1371/journal.pone.0276895

**Published:** 2022-10-27

**Authors:** Asma M. Karami, Yajiao Duan, Per W. Kania, Kurt Buchmann

**Affiliations:** Laboratory of Aquatic Pathobiology, Department of Veterinary and Animal Sciences, Faculty of Health and Medical Sciences, University of Copenhagen, Frederiksberg, Denmark; Veterinary Medical Research Institute, HUNGARY

## Abstract

Marker-assisted selective breeding of fish with higher levels of resistance towards specific pathogens may improve fish health, but the impact of host genotype on susceptibility to multiple pathogen infections is still poorly investigated. This study examined the resistance in rainbow trout *Oncorhynchus mykiss* towards infection with the eye fluke *Diplostomum pseudospathaceum*. We used genetically selected rainbow trout, carrying SNPs associated with resistance towards the parasitic ciliate *Ichthyophthirius multifiliis*, and exposed the fish to eye fluke cercariae. We showed that fish partly resistant to *I*. *multifiliis* were more susceptible to eye fluke invasion. The expression of immune relevant genes (encoding innate and adaptive factors) was also affected as these genotypes responded less strongly to a secondary fluke infection. The complexity of genome architecture in disease resistance towards multiple pathogens is discussed.

## Introduction

Genetic variation in teleosts is clearly associated with susceptibility/resistance to parasitic diseases. Models focusing on salmon louse infections [1], amoebic gill disease [2], white spot disease [3] and eye fluke invasion [4] point to a genetic background for parasitic disease resistance, aligning with previous findings on diseases caused by other pathogen types [5, 6]. The application of molecular markers for dissecting the genetic architecture associated with disease resistance allows the description of quantitative trait loci (QTL) [7], which can be used for breeding programs. This approach was applied for QTL searches in the rainbow trout genome pointing to chromosome 9 (Omy9) for whirling disease [8], Omy16 and Omy17 for White spot disease [9], Omy16 for furunculosis [10], and Omy21 for vibriosis [11]. The genetic background for resistance to multiple pathogen infections in fish is less clear. A positive genetic correlation with regard to different bacterial infections among full-sib families of Atlantic salmon or rainbow trout has been described. However, such an association is generally not seen between bacterial and viral infections [12, 13]. Only in a few cases, a weak positive genetic correlation between resistance to bacterial and viral infections may be seen [14], but in other cases, a strain strongly resistant to one pathogen may be entirely susceptible to another [15–17]. Partial cross-protection against the skin parasitic ciliate *Ichthyophthirius multifiliis* was noted in *Gyrodactylus derjavini* (a skin parasitic monogenean) immunized rainbow trout [18], suggesting that the same immune mechanism may be involved in natural resistance towards both parasitic pathogens, which attack the mucosal surfaces of the fish. However, the response mechanisms may differ between different types of parasites. Cercariae of the digenean eye fluke parasite *Diplostomum pseudospathaceum* penetrates the surface of the rainbow trout, shed the tail and migrate, as a diplostomules, in the vascular system of the fish, targeting the eye lens of the host. Such an infection route is likely to mainly involve systemic responses and challenge other parts of the host protection system. We have elucidated this question experimentally by exposing fish, carrying SNPs associated with *I*. *multifiliis* resistance, to *D*. *pseudospathaceum* cercariae and compared the infection outcome in fish with or without these SNPs. The *I*. *multifiliis* resistant fish were produced based on the QTL for reduced infection intensity and delayed time to death post-infection [9, 19]. We here present the eye fluke infection levels in the two rainbow trout strains following primary and secondary exposure to cercariae. In addition, we describe the expression of innate and adaptive immune genes in the fish and discuss how the different biology of the two parasite types may explain the differences found. Thus, the parasitic ciliate *I*. *multifiliis* has a direct life cycle and resides for more than a week in the epidermis of gills, fins and skin [20], whereas the digenean trematode, *Diplostomum pseudospathaceum*, has a more complex life cycle. The cercariae are in contact with the fish surface very short time during the penetration process. Diplostomules migrates within a few hours to days towards the eye lens, an immune-privileged host site, where the metacercarial stage is reached [21, 22]. We here show that rainbow trout, genetically selected for *I*. *multifiliis* resistance (QTL fish), obtain a higher eye fluke burden compared to fish heterozygous or negative for *I*.*multifiliis* resistance (non-QTL fish). The nature of the host defence mechanisms towards different parasitic infections are discussed.

## Materials and methods

### Ethics statement

The experiment was performed under license 2020-15-0201-00724 surveyed by the Experimental Animal Inspectorate, Committee for Experimental Animal, Ministry of Environment and Food, Denmark. It was reviewed by the Animal Ethics Institutional Review Board of the Faculty of Health and Medical Sciences at the University of Copenhagen. The ethical guidelines followed included continuous observation of fish every second hour securing removal and euthanization of fish with any clinical signs. In this study the fish did not show any clinical signs. Sampling of fish for parasite examination and organ sampling included prior euthanization with an overdose (300 mg/L, immersion) of ethyl-3-aminobenzoate methane-sulfonate (MS222) (cat.no. A5040, Sigma-Aldrich, Denmark). All exposed fish were sacrificed similarly after the end of the experiment. The snail used for providing parasite was sacrificed by freezing after the end of the experiment.

### Fish

A total of 200 rainbow trout (body weight 14.3–17.7 g, body length 10.2–11.5 cm) were used for the study. Two groups of rainbow trout with high (QTL fish) and low (non-QTL fish) frequency of SNPs associated with *I*. *multifiliis* resistance [9], were hatched from eyed eggs in December 2020 at the disease free recirculated Bornholm Salmon Hatchery, Nexø, Denmark [19] and subsequently reared to the fingerling stage. For this purpose, the first group (QTL-fish) was produced by using sperm from three male genotyped parents carrying SNPs AX-89947214 (Omy17) and AX-89960822 (Omy16), and the other group (non-QTL fish) was produced by using sperm from three other male parents negative for these SNPs. In both cases, sperm was used to fertilize a common pool of eggs stripped from a total of 30 outbred females. Processes of hatching and subsequent rearing of fry to the fingerling stage did not differ between groups of QTL fish and non-QTL fish [19]. From each group (QTL and non-QTL fish) we randomly gathered 100 rainbow trout and transported them (3 h duration) from the hatchery to the infection facility at the University of Copenhagen (May 2021). Fish were then accommodated and acclimatized 14 d in identical aerated glass tanks with internal biofilters (Eheim, Germany) (25 fish per 60 L water), which were placed in a temperature-controlled room (water temperature constant at 12°C, pH 7.6). We used 30% water exchange per day to maintain ammonia levels below 0.25 (AquaCheck, Hach, USA). Fish were fed by pelleted feed (Inicio, Biomar, Denmark, 1% of fish biomass per day).

### Parasites

In June 2021, we selected an infected *Lymnaea stagnalis* pulmonate snail (morphological identification according to Glöer [23] out of 205 snails collected in the lake Bagsværd sø (55°46’16.0566", 12°27’39.6864") (Zealand, Eastern part of Denmark). Snails were allocated in individual 100-ml beakers with filtered (0.45μm) lake water for subsequent shedding procedures [24]. All beakers were then examined under the stereomicroscope; among them, two snails shed diplostomid cercariae. These were preserved in ethanol (96%) for molecular identification, including PCR and sequencing, according to Duan et al. [24]. Isolated cercariae were identified as *D*. *pseudospathaceum* by morphology [25] and by sequencing of the internal transcribed spacer (ITS) region (rDNA). Only one of the two positive snails was highly productive and was selected for the experiment. It was maintained at room temperature in the beaker and fed fresh organic lettuce ad libitum. Water was replenished daily with filtered lake water. Freshly released cercariae were collected at regular intervals (3–4 h) and enumerated in Petri dishes under the dissection microscope. They were then added in equal amounts to the different fish tanks with fish.

### Experimental exposure

**Fig 1:**
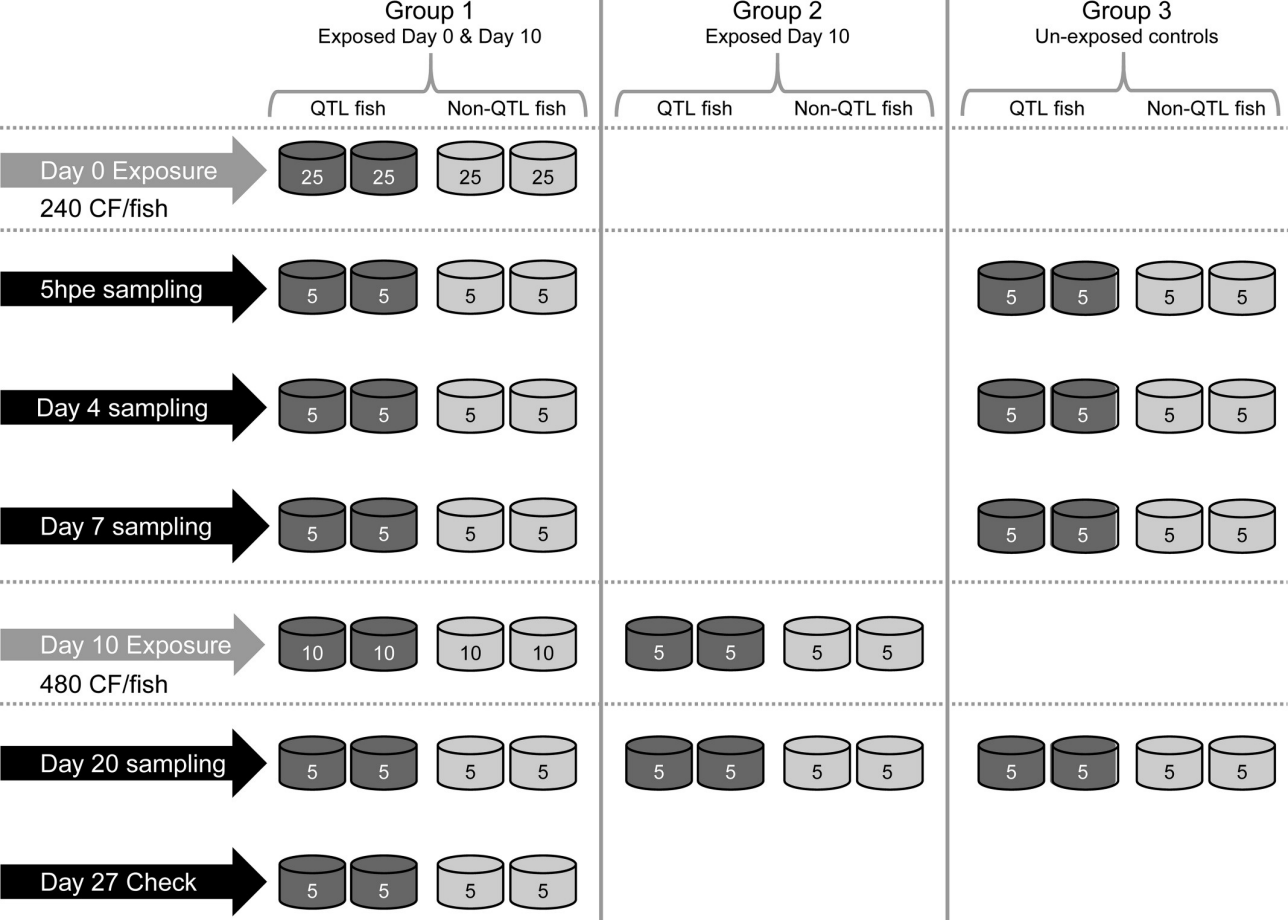
Experimental design of challenge study. At day 0, 4x25 fish were exposed to 240 cercariae per fish (C/F) constituting Group 1. At day 10, the remaining 4x10 of Group 1 and 4x5 naïve fish (Group 2) were exposed to 480 C/F. Group 3 was kept un-exposed throughout the entire experiment. At all samplings points, the number of metacercariae in the eyes was enumerated. At all sampling points except Day 27, gill and spleen from all fish were sampled for gene expression analyses and fins for genotyping.

Fig 1 outlines the experimental design. Exposure of fish to cercariae was conducted in duplicate for each group (QTL fish and non-QTL fish). Group 1. One hundred fish (50 QTL and 50 non-QTL fish) was exposed to 240 cercariae/fish during a 48 h period starting from day 0. Group 2. Twenty additional fish (10 QTL and 10 non-QTL) were kept un-infected until exposure on day 10, at which time the previously exposed fish (Group 1) also were exposed (re-exposure, see below). This group was applied as a control for the second exposure in Group 1. Group 3. Eighty fish (40 QTL-fish and 40 non-QTL fish) were kept as uninfected time-point control (not exposed) throughout the experiment.

Re-exposure (lasting 72 h) to cercariae (480 cercariae/fish) was initiated on day 10 for Group 1 and Group 2.

Sampling from euthanized fish comprised 2x5 fish per group per time-point. Sampled organs were eyes for enumeration of metacercariae, gill and spleen for gene expression and fins for genotyping. Sampling time-points were 5h post exposure (5hpe), 4, 7, 20 days post exposure (dpe) (Group 1 and 3) and 20 dpe for Group 2. An additional sampling was done for group 1 at 27 dpe (for enumeration of metacercariae only).

### Genotype confirmation

To approve the genetic difference between QTL and non-QTL fish, a 5 mm^2^ tail fin sample was taken from all fish. It was fixed in ethanol (96%), and stored for DNA extraction at 20°C. DNA extraction was performed following the manufacturer’s protocol of QIAamp® DNA Mini Kit (cat. No. 51306, Qiagen, Denmark). To amplify and subsequently sequence regions of discovered SNPs associated with white spot disease resistance [9], two primer sets were designed. SNP AX-89960822 (Omy16) was amplified / sequenced using forward primer OM_Chr 16 F2 (5’-CAAAGGCAGCACGATTGAGG-3’) and reverse primer OM_Chr 16 R1 (5’- ACATGTAAACACAGCGCTGG-3’) and for SNP AX-89947214 (Omy17) we used OM_Chr 17_F2 (5’- TCTGCTGTGTTGTGGGTGTT-3’) as forward primer and OM_Chr 17_R1 (5’-TTGCGGCTATGACTTGGAGG-3’) as reverse primer. PCR was conducted using a 60 μl reaction with 6 μl of purified genomic DNA. Each reaction contained 1 mM DNTP mix, 1 μM of each of forward and reverse primers, 1.5 mM MgCl_2_, 2 u BIOTAQ DNA Polymerase and supplemented with 6 μl 10x NH_4_-buffer (cat.no. Bio-2160, Saveen & Werner ApS, Denmark), and UltraPure™ DNase/RNase-Free Distilled Water (cat.no. 10977049, Thermo Fisher Scientific; Denmark) up to 60 μL. The PCR consisted of an initial denaturation step at 95°C for 5 min, 40 amplification cycles of denaturation at 95°C for 30s / annealing at 60°C for 30s /elongation at 72°C for 30s and a finally post elongation step at 72°C for 7 min. The obtained PCR products were analyzed by ethidium stained 2% agarose (cat.no. 10264544, Thermo Fisher Scientific, Denmark) gel electrophoresis, subsequently purified by illustra™ GFX™ PCR DNA and Gel Band Purification Kit (cat.no. 28-9034-71, VWR International A/S, Denmark) and send to Macrogen Europe B.V., Netherlands for sequencing. CLC workbench 20 was used to assemble the sequences.

### Enumerating metacercariae in fish

The number of metacercariae in eye lenses of the exposed and control fish was counted in all sampled fish by opening the eye and examining lenses and corpus vitreum under the dissection microscope (6–63X magnification). The mean intensity of infection (the number of metacercariae recorded in the fish divided by the number of infected fish) was calculated according to Bush et al. [26]. Infection success was calculated for each group and expressed as the percentage of cercariae used for exposure that managed to establish as metacercariae in the lenses adapted from Hoglund and Thuvander [27].

### Gene expression

Gill and spleen were sampled from each fish (QTL and non-QTL) in Groups 1, 2 and 3 at each time-point (5hpe, 4dpe, 7dpe and 20dpe). Sampled organs were fixed in RNAlater (cat.no R0901, Merck Life Science ApS, Denmark), placed at 4°C for 24h and subsequently stored at -20°C until further processing. Quantitative RT-qPCR were applied based on Karami et al. [11]. In brief, Tissue-lyser II, Qiagen, 20Hz, 2min were used for homogenization with 2-mercaptoethanol. Total RNA purification was done by the GenElute^TM^ mammalian RNA kit (cat. No. RTN250, Merck Life Science ApS, Denmark). DNase (AMPD1, Merck Life Science ApS, Denmark) treatment was applied to all samples, and RNA quality and quantity were determined by electrophoresis and NanoDrop 2000 spectrophotometer, respectively. Production of cDNA was done from 1000 ng of RNA with Oligo d(T)16 primer and MultiScribe^TM^ reverse transcription reagent (cat.no. 4311235, Thermo Fisher Scientific, Denmark); samples with no transcriptase were included as negative controls. AriaMx Real-Time PCR machine (AH diagnostics AS, Denmark) were used for gene expression analyses. Primers and Taq-Man probes used are listed in S1 Table. Reaction volumes were 12.5 μL [2.5 μl cDNA, 6.25 μl Brilliant III Ultra-Fast QPCR Master Mix (600881, AH Diagnostics AS, Denmark), 1.0 μL primer-probe mixture (forward primer, 10 μM and reverse primer, 10 μM), Taq-Man probe (5 μM), and 2.75 μl RNase-free water]. The running reactions temperature and process combination were: 95°C for 15 min, 45 cycles of denaturation at 94°C for 10s with a combined annealing/elongation process at 60°C for 45s. Elongation factor (ELF)1-α, β-actin and acidic ribosomal phosphoprotein P0 (ARP) were used as endogenous control and evaluated by NormFinder [28]. Gene expression data from this study have been deposited in NCBI’s Gene Expression Omnibus (GEO) and are accessible through GEO series accession number GSE213738.

### Statistics

To compare clearly different genotypes (QTL and non-QTL fish) we compared fish with a higher frequency of the favourable SNP (homozygous for the allele at least for one chromosome 16 or 17) to fish with a lower frequency of the favourable SNP (missing the favourable SNPs on one or both chromosomes). Double heterozygote fish were censored out from all analysis (see results).

To estimate the percentage of metacercariae reaching the eyes after the re-exposure at day 10, the mean intensity at 7dpe was subtracted from the re-exposed fish at 20 and 27dpe. One-way ANOVA (GraphPad Prism v.9.3.1) was applied to compare the mean intensities of metacercarial infection and the infection success between groups at each time point.

The relative gene expression analysis was based on the 2^−ΔΔCt^ method [29, 30], as all qPCR assays had efficiencies within 100% ± 5%. Two-way ANOVA was performed by GraphPad Prism v.9.3.1 to assess the influence of exposure (number of metacercariae) and genotype (QTL or non-QTL) (independent variables) on gene expression (dependent variable) and calculate the potential interaction between these variables. Significant regulation was defined when fold change was at least 2 and P<0.05.

## Results

### Genotype confirmation

Seven different genotypes may result from using a breeding strategy with homozygous males with respect to both of the favourable SNPs on Omy16 and Omy17 and combining these with wild-type females. This was in fact the result of the genotyping (Table 1). As expected, only fish predefined as QTL-fish were homozygous with respect to the favourable SNPs and none were homozygous with respect to the non-favourable SNPs. This was reversed in the non-QTL fish. However, both predefined types of fish comprised some double heterozygotes. These fish could not clearly be defined as QTL fish or non-QTL fish. To compare fish with a higher frequency of the favourable SNPs (at least 3) to fish with a lower frequency of the favourable SNPs (at most 1), the 37 double heterozygous fish were censored out. The GenBank accession numbers related to Omy16 are OP487875 to OP488054 and for Omy17 are OP488055 to OP488234.

**Table 1:** Distribution of combined genotypes. The number of fish with a specific genotype and its percentage in the sampled population is shown. The upper and lower cases indicate favourite and non-favourite nucleotides related to discovered SNPs on Omy16 and Omy17. T and G are the favourite SNPs on Omy16 and Omy17, respectively. Thus, the first and last columns of genotype show the number of fish with double homozygote for favourite and non-favourite SNPs. The middle column (Tg-Ga) shows the double heterozygotes, the only genotype present in both QTL and non-QTL fish.

	Genotype (Omy16-Omy17)						
	TT-GG	TT-Ga	Tg-GG	Tg-Ga	gg-Ga	Tg-aa	gg-aa
QTL	23	8	45	14	0	0	0
	25.6%	8.9%	50.0%	15.6%			
Non-QTL	0	0	0	23	38	12	17
				25.6%	42.2%	13.3%	18.9%
All	23	8	45	37	38	12	17
	12.8%	4.4%	25.6%	20.0%	21.1%	6.7%	9.4%

### Enumerating metacercariae in fish

**Fig 2:**
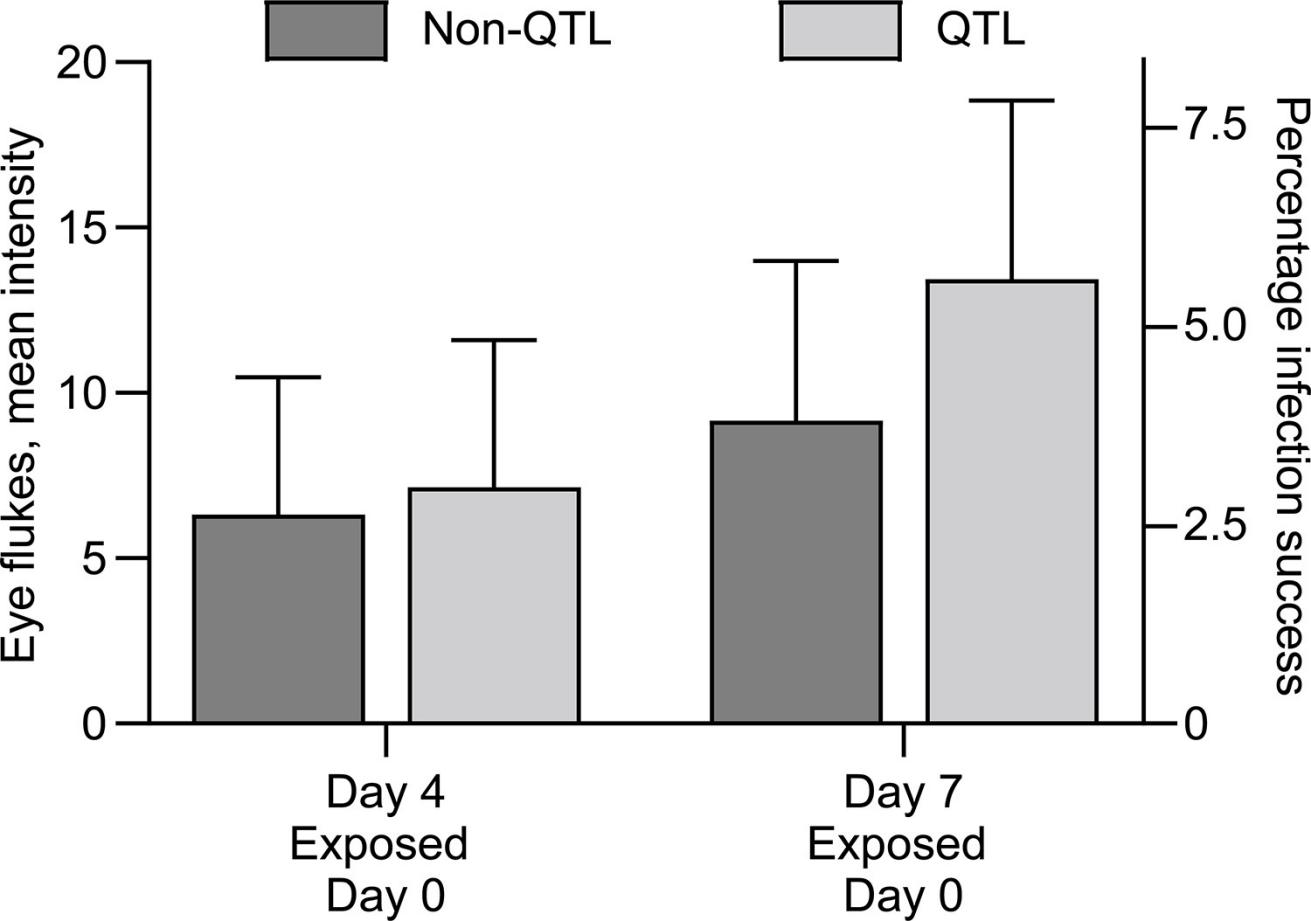
Eye fluke mean intensity and infection success in QTL and non-QTL fish on 4 and 7 days post exposure.

**Fig 3:**
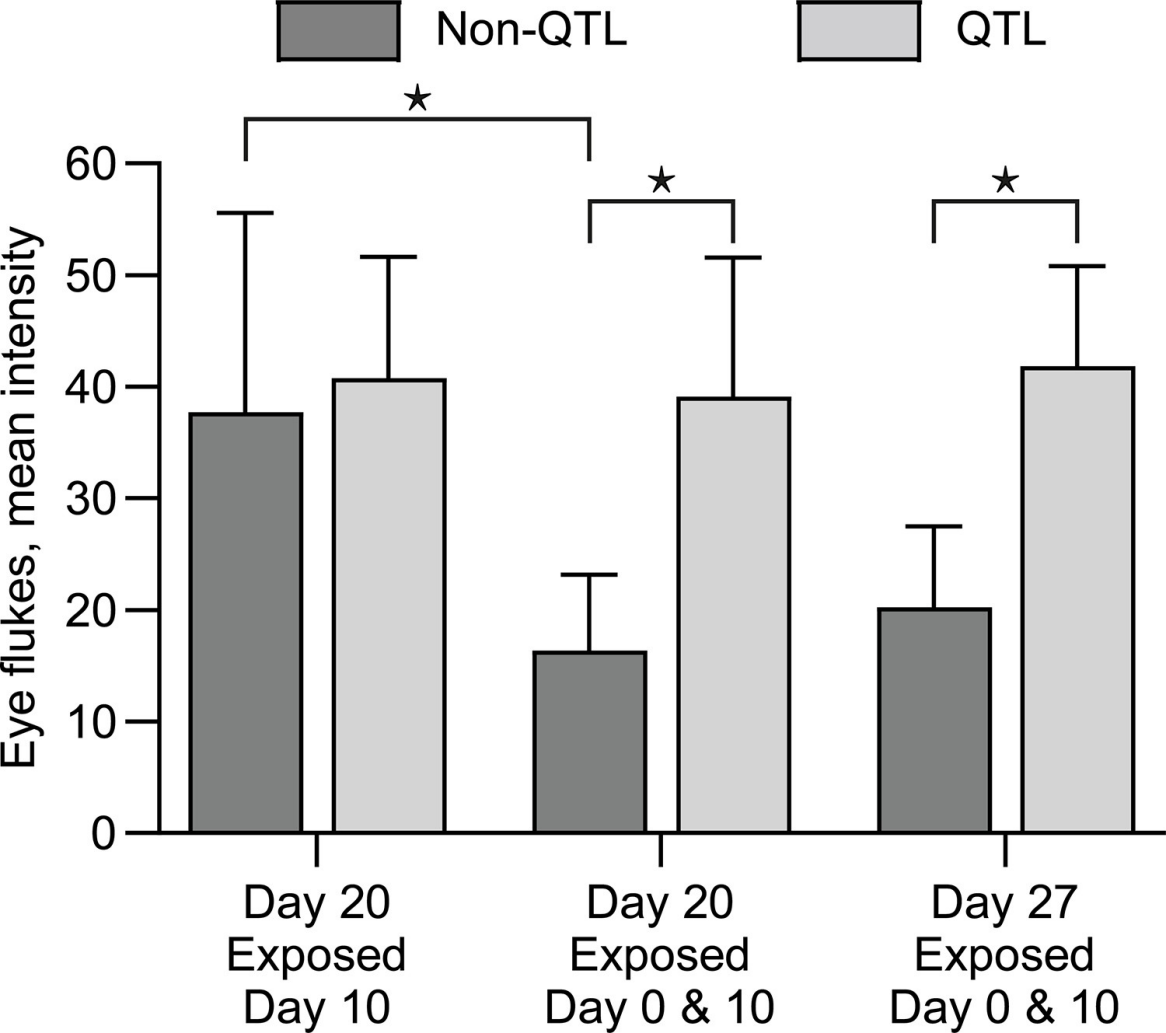
Eye fluke mean intensity in QTL and non-QTL fish on 20 and 27 days post exposure for Group 1 (exposed on days 0 and 10) and Group 2 (exposed only on day 10). *: p<0.05.

Control fish (non-exposed) did not carry any metacercariae in their eyes (QTL and non QTL). No metacercariae were detected in any fish found at 5hpe. The mean intensities in QTL and non-QTL fish exposed to cercariae were compared on 4 and 7 dpe. The mean intensity of metacercarial infection in QTL fish was higher on 7dpe compared to non-QTL fish (Fig 2). When we at 20dpe compared Group 1 (exposed on days 0 and 10) to Group 2 (exposed only on day 10) we recorded a significantly lower load of metacercariae in non-QTL fish (Fig 3). Enumerating metacercariae on 27dpe in Group 1 confirmed the higher load of the parasite in QTL fish compared to non-QTL more than three weeks after the first exposure (Fig 3). The re-exposure at day 10 resulted in a significantly lower infection in primed fish (Group 1) compared to naïve fish exposed at the same time (Group 2) when examined at 20dpe. Statistics showed the same differences among the groups when comparing the infection success percentage. Mean intensities and infection success rates are shown in Table 2.

**Table 2:** Mean intensity (the average number of metacercariae divided by the number of infected fish examined in the sample) and Infection success (the percentage of cercariae that managed to establish as metacercariae in the lens) are shown for QTL and non-QTL fish in 5hpe, 4dpe, 7dpe and 20 dpe fish Group 1 (exposed on days 0 and 10) and Group 2 (exposed on day 10) and 27dpe fish Group 1.

	5hpe		4dpe		7dpe		20dpe—Group1		20dpe–Group 2		27dpe—Group 1	
	Mean intensity	Infection success	Mean intensity	Infection success	Mean intensity	Infection success	Mean intensity	Infection success	Mean intensity	Infection success	Mean intensity	Infection success
QTL-Fish	0	0	7.1	3%	13.4	5.6%	38.9	5.3%	40.8	8.5%	41.9	5.9%
Non-QTL Fish	0	0	6.3	2.6%	9.2	3.8%	16.4	1.5%	37.7	7.9%	20.3	2.3%

### Gene expression

**Fig 4:**
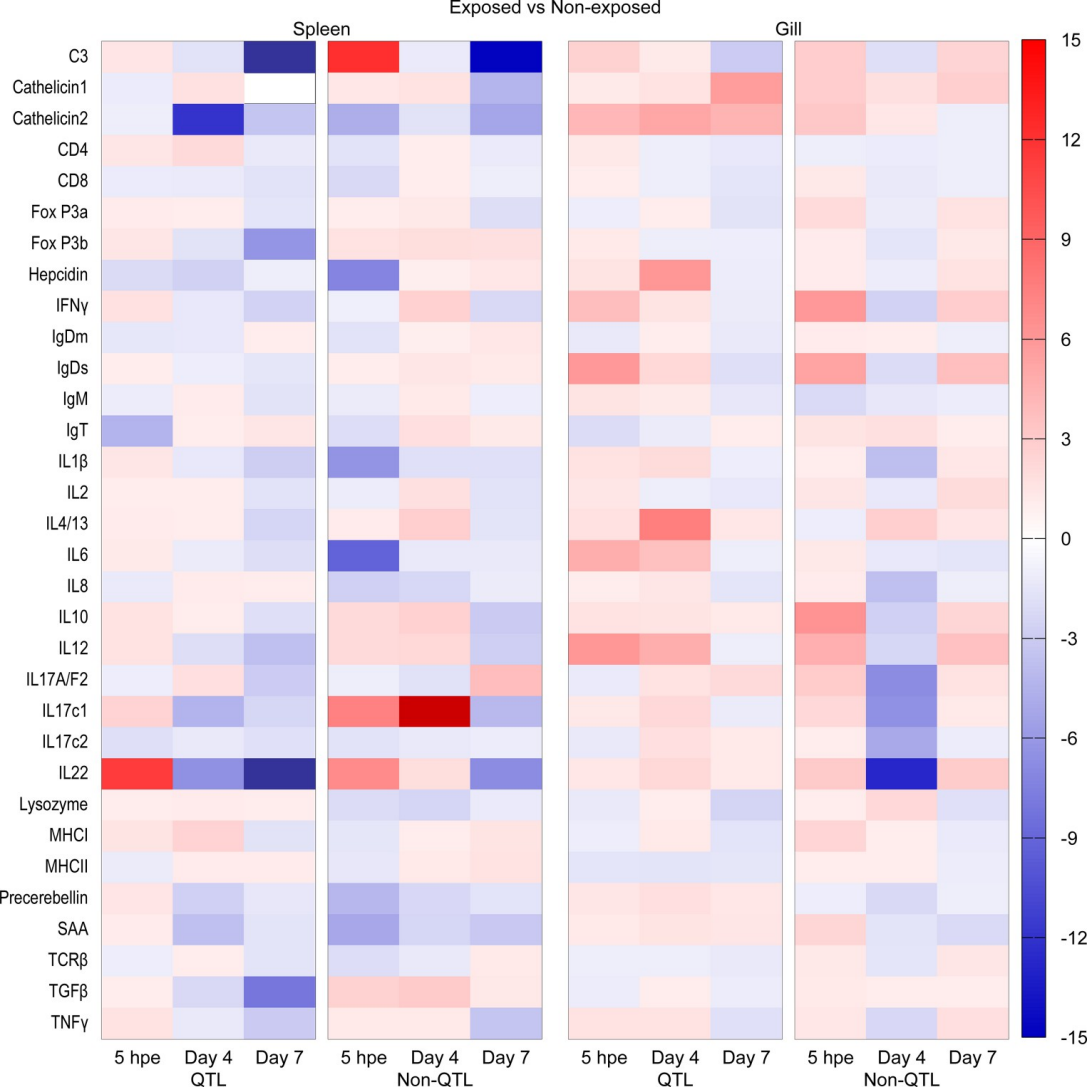
Overview of gene expression in gill and spleen of QTL and non-QTL fish on 5h, 4 and 7 days post exposure.

**Fig 5:**
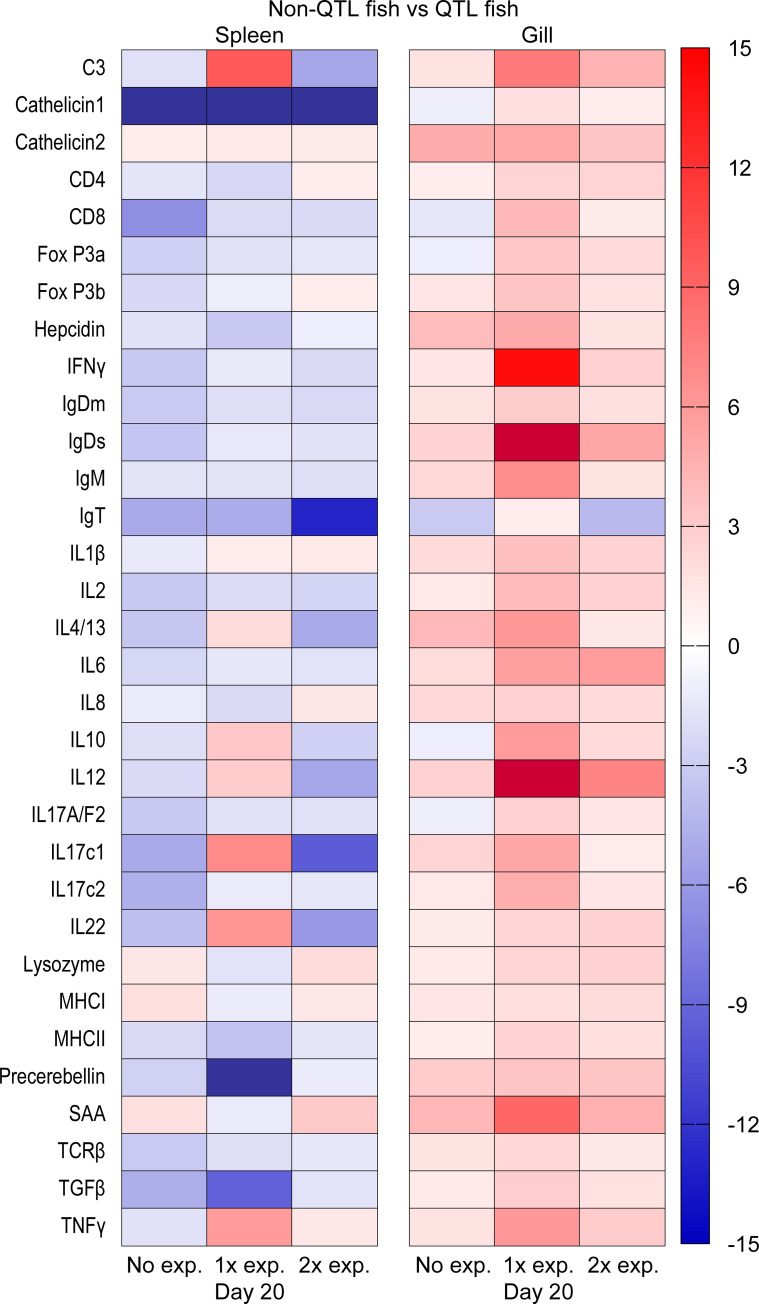
Overview of gene expression in gill and spleen of QTL and non-QTL fish on 20 days post exposure for Group 1 (exposed on days 0 and 10) and Group 2 (exposed only on day 10).

Details on all genes and their regulation including fold changes and p values are shown in S2 Table and S1 Fig. For an overall view, heat maps were generated based on fold changes between control and exposed fish (Fig 4) and non-QTL and QTL fish (Fig 5).

A general expression pattern was seen in non-exposed control fish. First of all the gills and spleen showed a different constitutive expression. When comparing the expression of 32 genes in control fish (non-exposed) more genes were regulated in the gill (23 genes) than in the spleen (14 genes). Also, the two genetic strains were different. Comparison between QTL and non-QTL fish (non-exposed) showed higher expression in non-QTL fish in gill (except for genes encoding IL10 (5hpe) and IgT (4dpe)) but downregulation in the spleen (except for genes encoding Hepcidin, IL-1β, IL-8, MHCI and SAA at 5hpe).

In the gill of exposed QTL fish (Group 1) compared to non-exposed fish, we found a significant upregulation in genes encoding Cath 2 (5hpe, 4 and 7 dpe), IFNγ, IgDs, IL-6 (5hpe), Hepcidin, IL-1β, IL-4/13 and IL-12 (4dpe). The gene expression overview in QTL fish shows an early regulation (5hpe) which gradually decreased during infection or reached a peak on 4dpe and dropped again on 7dpe. Only a few genes (Cath1, Cath2, IgT and IL17A/F2) showed upregulation on 7dpe (Fig 4).

In the gill of non-QTL fish (Group 1), 17 genes showed significant upregulation when compared to control (non-exposed) fish. These genes encoded C3, Cath2, Fox P3a, IFNγ, IgDs, IL-1β, IL-2, IL-4/13, IL-10, IL-12, IL-17A/F2, IL-17c1, IL-17c2, IL-22, Precerebellin, SAA and TNFαeither on 5hpe or 7dpe or both time points. It is worth mentioning that the majority of the examined genes showed upregulation on 5hpe followed by a downregulation on 4dpe with a subsequent rise on 7dpe (Fig 4).

Comparing fold change between infected QTL and non-QTL fish gill in different time points showed a significant upregulation in non-QTL fish for genes encoding C3, Fox P3a, IFNγ, IgDs, IgM, IL-2 and IL-12 on 7dpe (S2 Table and S1 Fig).

In the spleen of control fish (QTL compared to non-QTL), most of the examined genes were downregulated except for those encoding Hepcidin, IL-1β, MHCI and TNFα (S2 Table and S1 Fig).

The overview of the gene expression in the spleen of infected QTL and non-QTL fish compared to control showed no significant regulation for most of the examined genes.

Comparing gene expression in the spleen of infected QTL and non-QTL fish showed significant downregulation in QTL fish for most of the genes except for MHCI, which was 4 times higher in non-QTL fish (S2 Table and S1 Fig).

In the re-exposure study, comparing gill from QTL and non-QTL fish Group 1 (exposed on days 0 and 10) showed significant upregulation in genes encoding C3, Cath2, CD4, IgDs, IL-1β, IL-2, Lysozyme, Precerebellin, SAA and TNFα in non-QTL fish. Upregulation of genes encoding IFNγ, IgM and IL4/13 were significantly higher in non-QTL fish from Group 2 (exposed on day 10). Comparing spleen from QTL and non-QTL fish from Group 1 and Group 2 showed no significant changes for most of the genes except upregulation in the gene encoding Fox P3a in non-QTL fish (Figs 4 and 5).

## Discussion

Two main questions were addressed in this study. First, we wished to elucidate if fish showing a level of resistance to infection with the skin parasitic *I*. *multifiliis* (QTL fish) also showed some protection against eye fluke *D*. *pseudospathaceum* infections. Secondly, we investigated if previous exposure to eye fluke invasion (both QTL and non-QTL fish) induced some response to a secondary fluke infection.

Surprisingly, rainbow trout carrying SNPs associated with a lower *I*. *multifiliis* infection and delayed time to death, as described by Jaafar et al. [9] and Buchmann et al. [19], were more susceptible to eye flukes and obtained a significantly higher parasite load in the lenses. This was most clearly shown following re-exposure to eye fluke cercariae. This suggests that the factors protecting fish against *I*. *multifiliis* differ from mechanisms protecting trout against eye flukes.

We demonstrated that, in both QTL and non-QTL fish, the infection intensity increases during exposure to infective cercariae. This corresponds to the dynamics shown in natural lakes and at farm level [31, 32]. However, the second exposure resulted none-the-less in a slightly but significantly lower infection success, which suggests that a previous infection confers a relative protection to re-infection. This was previously shown in other experimental and field studies [27, 22, 33, 34]. The protective mechanisms in repeatedly eye fluke exposed fish was suggested to include both avoiding behaviour and elements of acquired immunity [22, 33]. According to Hoglund and Thuvander [27], the response is primarily based on innate immune factors because the authors did not confirm changes in specific antibody titers over the infection course. They showed that various leukocytes (neutrophils and monocytes/macrophages) were recruited in primed fish, suggesting that cellular factors are involved. In this context, it is noteworthy that Chappel [35] also suggested that the immune reaction involved an elevated activation of macrophages, through TNFα and IFNγ upregulation, with resulting ROS (reactive oxygen species) production. Direct killing of diplostomules and subsequent phagocytosis during the migration in the host before reaching the eye would then explain the partial protection.

In the present study, we confirmed that eye fluke infection elicited upregulation of genes encoding cytokines like IL-1β, IFNγ, IL-6 and IL-12. This aligns with the suggested immune pathway in stickleback responses towards *Diplostomum* invasion [4, 36, 37], again pointing at elevated monocyte proliferation and respiratory burst activity.

We recorded a general parasite-induced upregulation of genes encoding immunoglobulins (IgM and IgD) in exposed fish gills. Such an immunoglobulin involvement could be interpreted as a humoral response in the interface between innate and adaptive reactions. Although specific antibody titers may not result from the process, this local response aligns with elevated production of low specificity serum antibodies in *Diplostomum* exposed rainbow trout [27, 38]. If the low specific antibody level is due to antigen disguise and or antigen shifts during the diplostomule migration is worth investigating in the future. Alternatively, the low antibody production might because of the fast migration of diplostomules to the eye, limiting the time for humoral response. It is also noteworthy that IgD, as shown in the present study, seems to play a prominent role in the early response to infection. This suggests that new IgD antibody assays should be developed to evaluate if the specificity of IgD antibodies is higher than found for IgM.

We also demonstrated a significant regulation of the gene encoding Complement Factor C3 in the gill and spleen supporting the view that serum factors is part of the innate response [35, 36]. These innate serum factors may directly kill the pathogen or opsonize parasite surfaces and thereby improve leucocyte adherence to the invader.

The present study showed a higher level of major histocompatibility complex (MHC) transcripts in infected fish. MHC molecules, especially Class II, present peptides from extracellular parasites. Allelic diversity of these molecules have been suggested to increase the immune response efficiency [39], although Rauch et al. [40] suggested that other genes influence the parasite load more than MHC allelic diversity. However, macrophages (involved in both innate and adaptive responses) carry these molecules, and it is likely that both immune pathways are activated during the short migration of diplostomules to the immune-privileged fish lenses. Repeated exposure to cercariae resulted in a slightly lower infection success rate, which may due to a higher trapping/elimination of diplostomules by the humoral and cellular innate elements in the body of these primed fish.

We found a significant difference in the number of metacercariae in lenses of QTL and non-QTL fish which suggests a genetic background for eye fluke resistance. This genetic aspect was previously indicated [41–43], but in this study we saw that the mechanisms differed clearly from the ones associated with *I*. *multifiliis* resistance. Thus, the QTL rainbow trout in this study were produced by use of male parent fish homozygous for the favourite SNPs on Omy16 and 17 associated with a relative resistance (lower intensity of infection and delayed onset of mortality) to *I*. *multifiliis* infection [9, 19]. Our approach of exposing genotyped rainbow trout resistant to *I*. *multifiliis* to another parasite, *Diplostomum*, could elucidate if the genes involved in protection against a parasitic ciliate are relevant for other parasite types. The host response against the parasitic ciliate, *I*. *multifillis*, in rainbow trout involves both innate and adaptive immune factors, such as neutrophil activity [44], upregulation of genes encoding antimicrobial peptides, complement factors and serum amyloid A [9] supplemented by adaptive immunity elements involving IgM, IgT and T cell responses [45].

Although classical immune factors may play a role in the genetic basis for inherited resistance to parasite infection, other physiological factors (in parasite and host) should be discussed. *I*. *multifillis* theronts are attracted to host serum molecules released from mucous cells, and the rapid penetration of theronts into the fish epidermis probably occurs through mucous cell openings [18, 46]. Therefore, it is possible that chemo-attraction of *I*. *multifiliis* to the host plays a role in the inheritable susceptibility/resistance to a primary infection and that humoral and cellular play a role in response to a secondary infection–both for *Ichthyophthirius* and *Diplostomum*.

As these specialized chemoattractive elements may be involved in the susceptibility of rainbow trout to *I*.*multifillis* infection, it is still possible that a multicellular parasite, such as *Diplostomum*, may apply other host finding mechanisms resulting in a different infection success compared to *I*. *multifillis*. Dissection of the rainbow trout genome close to SNPs located on Omy16 may shed light on the involved genes. Here one finds a gene encoding a mucin-1-like protein, an O-glycosylated glycoprotein belonging to membrane-bound mucins family [47]. Certain specific molecular structures of mucin or mucous cells may, at least theoretically, affect the vulnerability of QTL fish to *I*. *multifillis*. Correspondingly, *Diplostomum* could be affected differently.However, involvement of at least a partial role of the mucous surfaces, with regard to eye flukes, cannot be excluded as these factors were affected in e.g. common carp by feeding beta-glucan, nucleotides or chitosan which reduced the susceptibility to *Diplostomum* [48].

For both parasites innate and adaptive host immune responses do play a role. Toll-like receptors (TLRs) recognize these parasites invading the epithelium of the skin, gill, and other body surfaces. Proinflammatory responses are then stablished, as judged by the release of cytokines (IFNɣ, IL-1β), which attract neutrophils and monocytes to the infection area. IgT and IgM binding and recruitment of CD8^+^ cells and MHCII^+^ cells in mucosal and systemic tissues are involved in both cases. Thus, for both types of parasites, we suggest that different phases of the infection process should be discerned when discussing susceptibility and resistance. The process can be divided into 1) chemoattraction of a parasite to the host, 2) the attachment of the parasite to the host, 3) the penetration of the host surface, and 4) subsequent survival in the host organism. The ability of the parasite to down-regulate central immune reactions, which are potentially lethal to invading parasite, is likely to differ between different species and strains of both hosts and parasites. Future studies should therefore elucidate the four factors in further depth.

## Supporting information

S1 Data (XLSX)

S1 Table List of primers and probes. (DOCX)

S2 Table Gene expression results. (XLSX)

S1 Fig Gene expression results. A. Gene expression result. First part of cytokine genes. The gene expression assessed using Two Way ANOVA. Significant regulation defined as: fold change at least 2 and p<0.05. *: p<0.05. Source of variation (p<0.05) is indicated by letters in circles above; G indicates Genotype; E indicates Exposure; I indicates Interaction between Genotype and Exposure. The y-axis represent geometric means and error bars geometric standard deviations. B. Gene expression result. Second part of cytokine and transcription factor genes. The gene expression assessed using Two Way ANOVA. Significant regulation was defined as both fold change was at least 2 and p<0.05. Asterisks indicate p<0.05. Source of variation (p<0.05) is indicated by letters in circles above; G indicates Genotype; E indicates Exposure; I indicates Interaction between Genotype and Exposure. The y-axis represent geometric means and error bars geometric standard deviations. C. Gene expression result. Immune cell marker genes. The gene expression assessed using Two Way ANOVA. Significant regulation defined as: fold change at least 2 and p<0.05. *: p<0.05. Source of variation (p<0.05) is indicated by letters in circles above; G indicates Genotype; E indicates Exposure; I indicates Interaction between Genotype and Exposure. The y-axis represent geometric means and error bars geometric standard deviations. D. Gene expression result. One marker for immune cells and 7 innate factors. The gene expression assessed using Two Way ANOVA. Significant regulation defined as: fold change at least 2 and p<0.05. *: p<0.05. Source of variation (p<0.05) is indicated by letters in circles above; G indicates Genotype; E indicates Exposure; I indicates Interaction between Genotype and Exposure. The y-axis represent geometric means and error bars geometric standard deviations. E. Gene expression result of the re-exposure trial. Only the 24 genes significantly regulated are shown. The gene expression assessed using Two Way ANOVA. Significant regulation defined as: fold change at least 2 and p<0.05. *: p<0.05. Source of variation (p<0.05) is indicated by letters in circles above; G indicates Genotype; E indicates Exposure; I indicates Interaction between Genotype and Exposure; results for spleen an gill are placed to the left and right, respectively. Please note the figure focus on the genotypes non-QTL fish versus QTL fish. The y-axis represent geometric means and error bars geometric standard deviations.
(PDF)
