# Supplementary material for: Responses towards eyefluke (Diplostomum pseudospathaceum) in different genetic lineages of rainbow trout
Source: PLoS One. 2022 Oct 27;17(10):e0276895. doi: 10.1371/journal.pone.0276895 (PMC9612474; doi:10.1371/journal.pone.0276895)
Supplement: S1 Table — (DOCX) [file pone.0276895.s002.docx]

**Table S1**

Primers and probes used for qPCR assays. All nucleotides are read from 5’ end to 3’end. The probes are labelled with FAM at the 5’end and BHQ1 at the 3’ end. The qPCR assays were all optimized to have annealing temperature of 60°C and efficiencies of 100% ± 5%. ^R^ indicates reference genes (housekeepers). ^MS^ indicates the qPCR assay targets both membrane bound and secreted forms. ^1^ The α chain of IL-12 is common to the two isoforms of IL-12.

| **Gene**  **GenBank acc.no.** | **Length**  **bp** | **Primers & Probes**  **5’end to 3’end** | **References** |
| --- | --- | --- | --- |
| ^R^ ARP  AY505012 | 106 | Fwd: GAAAATCATCCAATTGCTGGATG  Rev: CTTCCCACGCAAGGACAGA  Probe: CTATCCCAAATGTTTCATTGTCGGCGC | Purcell, Kurath, Garver, Herwig and Winton (2004) |
| ^R^ β-actin  AB196465 | 241 | Fwd: ACATCAAGGAGAAGCTGTGCTAC  Rev: TACGGATGTCCACGTCACAC  Probe: CCTCTCTGGAGAAGAGCTACGAGCTG | Marana, Chettri, SaltenBach-Olesen, Kania, Dalsgaard and Buchmann (2020) |
| ^R^ ELF-1α  [AF498320](http://www.ncbi.nlm.nih.gov/entrez/viewer.fcgi?db=nucleotide&val=20269865) | 63 | Fwd: ACCCTCCTCTTGGTCGTTTC  Rev: TGATGACACCAACAGCAACA  Probe: GCTGTGCGTGACATGAGGCA | Ingerslev, Pettersen, Jakobsen, Petersen and Wergeland (2006) |
| C3.3 & C3.4  AF271080 / U61753 | 85 | Fwd: ATTGGCCTGTCCAAAACACA  Rev: AGCTTCAGATCAAGGAAGAAGTTC  Probe: TGGAATCTGTGTGTCTGAACCCC | Raida and Buchmann (2009) |
| Cathelicidin 1A  AY382478 | 189 | Fwd: TCTCTCGTCCTGGGGTT  Rev: GTTGTAGCGTGCTGATCTATG  Probe: TAATTGGTCGTCCTGGGGGTGG | Marana *et al.* (2020) |
| Cathelicidin 2A  AY360356 | 135 | Fwd: AAAGATTCCAAGGGGGGT  Rev: CAAAGGGTGTGTTGTGCTGT  Probe: GCTCTCGTCCTGGGTTTGGCTCC | Xueqin, Kania and Buchmann (2012) |
| CD4  AY973028 | 89 | Fwd: CATTAGCCTGGGTGGTCAAT  Rev: CCCTTTCTTTGACAGGGAGA  Probe: CAGAAGAGAGAGCTGGATGTCTCCG | Raida and Buchmann (2008b) |
| CD8a  AF178054 | 74 | Fwd: ACACCAATGACCACAACCATAGAG  Rev: GGGTCCACCTTTCCCACTTT  Probe: ACCAGCTCTACAACTGCCAAGTCGTGC | Olsen, M.M., Kania, P.W., Heinecke, R.D., Skjoedt, K., Rasmussen, K.J. & Buchmann, K. v2011) |
| Fox P3a  FM883710 | 80 | Fwd: CTACAGGCACAGCCTGTCACTAGG  Rev: GCTCCTCTGGCTCTTTAGTGG  Probe: CCAGAACCGAGGTGGAGTGTCACG | Bahlool QZ, Skovgaard A, Kania PW, Buchmann K (2013) |
| Fox P3b  FM883711 | 75 | Fwd: TCCTGCCCCAGTACTCATCCC  Rev: GCTCCTCTGGCTCTTTAGTGG  Probe: CTTGGCAGCAGATGGAGTGCCACG | Bahlool QZ, Skovgaard A, Kania PW, Buchmann K (2013) |
| Hepcidin | 95 | Fwd: GAGGAGGTTGGAAGCATTGA  Rev: TGACGCTTGAACCTGAAATG  Probe: AGTCCAGTTGGGGAACATCAACAG | Raida, M.K. & Buchmann, K. (2009) |
| IFN γ1 and IFN γ2  FJ184374 / FJ184375 | 68 | Fwd: AAGGGCTGTGATGTGTTTCTG  Rev: TGTACTGAGCGGCATTACTCC  Probe: TTGATGGGCTGGATGACTTTAGGA | Raida and Buchmann (2007) |
| IgDm  AY870262 | 304 | Fwd: CAGGAGGAAAGTTCGGCATCA  Rev: CCTCAAGGAGCTCTGGTTTGGA  Probe: CCACACCACACAGACTCTGGCCCTGAA | Skov, Chettri, Jaafar, Kania, Dalsgaard and Buchmann (2018) |
| IgDs  JQ003979 | 304 | Fwd: TGGCACGCCAGGATTTGAC  Rev: TCAGAATTGAGTGAACGGACAGACA  Probe: CCACACCACACAGACTCTGGCCCTGAA | Skov *et al.* (2018) |
| ^MS^ IgM  S63348 / AH014877 | 72 | Fwd: ACCCTCCTCTTGGTCGTTTC  Rev: TGATGACACCAACAGCAACA  Probe: TGATGACACCAACAGCAACA | Raida and Buchmann (2007) |
| ^MS^ IgT  AY870265 / AY870263 | 73 | Fwd: AGCACCAGGGTGAAACCA  Rev: GCGGTGGGTTCAGAGTCA  Probe: AGCAAGACGACCTCCAAAACAGAAC | Raida and Buchmann (2007) |
| IL1β  AJ223954 | 91 | Fwd: ACATTGCCAACCTCATCATCG  Rev: TTGAGCAGGTCCTTGTCCTTG  Probe: CATGGAGAGGTTAAAGGGTGGC | Raida and Buchmann (2007) |
| IL2a  FJ571513 | 110 | Fwd: ATGCAACACCACATCAGCAT  Rev: TGCCACGGCCCTACAAAAGA  Probe: TGCCACGGCCCTACAAAAGA  RE  TGCCACGGCCCTACAAAAGA | Marana *et al.* (2020) |
| IL4/13a  AB574337 | 138 | Fwd: ATCCTTCTCCTCTCTGTTGC  Rev: GAGTGTGTGTGTATTGTCCTG  Probe: CGCACCGGCAGCATAGAAGT | Chettri, Kuhn, Jaafar, Kania, Moller and Buchmann (2014) |
| IL6a  DQ866150 | 91 | Fwd: ACTCCCCTCTGTCACACACC  Rev: GGCAGACAGGTCCTCCACTA  Probe: CCACTGTGCTGATAGGGCTGG | Raida and Buchmann (2008a) |
| IL8 isoforms a, b , c, d & e  AY160982 to AY160986 | 69 | Fwd: AGAATGTCAGCCAGCCTTGT  Rev: TCTCAGACTCATCCCCTCAGT  Probe: TTGTGCTCCTGGCCCTCCTGA | Raida and Buchmann (2008a) |
| IL10a  [AB118099](http://www.ncbi.nlm.nih.gov/entrez/viewer.fcgi?db=nucleotide&val=47678892) | 70 | Fwd: CGACTTTAAATCTCCCATCGAC  Rev: GCATTGGACGATCTCTTTCTTC  Probe: CATCGGAAACATCTTCCACGAGCT | Raida and Buchmann( 2007) |
| ^1^ IL12 α chain  HE798148 | 84 | Fwd: CAACGGAACACCACATTCAG  Rev: AGCCTGTAGTGAGGCAGCAT  Probe: TGCGTGTCTGAGGAACATCCG | Jaafar, ChettriDalsgaard, Al-Jubury, Kania, Skov and Buchmann (2015) |
| IL17A/F2a  AJ580842 | 158 | Fwd: TCAAAAGCAACGTGTCGAAG  Rev: TCCCTCTGATTCCTCTGTGG  Probe: TATGCTGCTGGGCCTGACCA | Jaafar *et al.* (2015) |
| IL17c1  CAW30792 | 138 | Fwd: CTGGCGGTACAGCATCGATA  Rev: GAGTTATATCCATAATCTTCGTATTCGGC  Probe: CGTGATGTCCGTGCCCTTTGACGATG | Chettri *et al.* (2014) |
| IL17c2  CAW30793 | 134 | Fwd: CTGGCGGTACAGCATCGATA  Rev: CAGAGTTATATGCATGATGTTGGGC  Probe: CGTGGTGTCCAGGCCCTTTAATGATG | Chettri *et al.* (2014) |
| IL22  AM748537 | 64 | Fwd: ATGACCACCACCACAGCATT  Rev: ATTCCTTTCCCCTCCTCCAT  Probe: CTTTCCGCAAGAAGTTGTCCGAG | Olsen, Kania, Heinecke, Skjoedt, Rasmussen and Buchmann (2011) |
| Lysozyme  X59491 | 188 | Fwd: GAAACAGCCTGCCCAACT  Rev: GTCCAACACCACACGCTT  Probe: ATACCCAGGCCACCAACCGCAACAC | Chettri, Raida, Kania and Buchmann (2012) |
| MHC I  AY523661 | 73 | Fwd: TCCCTCCCTCAGTGTCT  Rev: GGGTAGAAACCTGTAGCGTG  Probe: CAGAAGACCCCCTCCTCTCCAGT | Skov, J., Kania, P.W., Holten-Andersen, L., Fouz, B. & Buchmann, K. (2012) |
| MHC II  AF115533 | 68 | Fwd: TGCCATGCTGATGTGCAG  Rev: GTCCCTCAGCCAGGTCACT  Probe: CGCCTATGACTTCTACCCCAAACAAAT | Raida, M.K. & Buchmann, K. (2007) |
| Precerebillin  AF192969 | 116 | Fwd: TGGTGTTGCTTTGCTGTTGT  Rev: TGGTGTTGCTTTGCTGTTGT  Probe: ATGGTTGAGACTCAGACGGAGAGTG | Raida, M.K. & Buchmann, K. (2009) |
| SAA  AM422446 | 79 | Fwd: GGGAGATGATTCAGGGTTCCA  Rev: TTACGTCCCCAGTGGTTAGC  Probe: TCGAGGACACGAGGACTCAGCA | Skov, Kania, Holten-Andersen, Fouz and Buchmann (2012) |
| TCR-β  AF329700 | 73 | Fwd: TCACCAGCAGACTGAGAGTCC  Rev: AAGCTGACAATGCAGGTGAATC  Probe: CCAATGAATGGCACAAACCAGAGAA | Raida and Buchmann (2007) |
| TGF-β1a  [X99303](http://www.ncbi.nlm.nih.gov/entrez/viewer.fcgi?db=nucleotide&val=1478246) | 75 | Fwd: TCTGAATGAGTGGCTGCAAG  Rev: GGTTTCCCACAATCACAAGG  Probe: CTGGAGAGGAGCAGGGATTCCAAT | Raida and Buchmann (2007) |
| TNF-α1 & TNF-α2  AJ277604 / AJ401377 | 75 | Fwd: GGGGACAAACTGTGGACTGA  Rev: GAAGTTCTTGCCCTGCTCTG  Probe: GACCAATCGACTGACCGACGTGGA | Raida and Buchmann (2008a) |

**References:**

Bahlool QZ, Skovgaard A, Kania PW, Buchmann K (2013). Effects of excretory/secretory products from Anisakis simplex (Nematoda) on immune gene expression in rainbow trout (Oncorhynchus mykiss). Fish Shellfish Immunol. 2013 Sep;35(3):734-9. doi: 10.1016/j.fsi.2013.06.007

Chettri, J.K., Kuhn, J.A., Jaafar, R.M., Kania, P.W., Moller, O.S. & Buchmann, K. (2014) Epidermal response of rainbow trout to Ichthyobodo necator: immunohistochemical and gene expression studies indicate a Th1-/Th2-like switch. *J Fish Dis,* **37,** 771-783.

Chettri, J.K., Raida, M.K., Kania, P.W. & Buchmann, K. (2012) Differential immune response of rainbow trout (Oncorhynchus mykiss) at early developmental stages (larvae and fry) against the bacterial pathogen Yersinia ruckeri. *Developmental & Comparative Immunology,* **36,** 463-474.

Ingerslev, H.-C., Pettersen, E.F., Jakobsen, R.A., Petersen, C.B. & Wergeland, H.I. (2006) Expression profiling and validation of reference gene candidates in immune relevant tissues and cells from Atlantic salmon (Salmo salar L.). *Molecular Immunology,* **43,** 1194-1201.

Jaafar, R.M., Chettri, J.K., Dalsgaard, I., Al-Jubury, A., Kania, P.W., Skov, J. & Buchmann, K. (2015) Effects of adjuvant Montanide™ ISA 763 A VG in rainbow trout injection vaccinated against Yersinia ruckeri. *Fish & Shellfish Immunology,* **47,** 797-806.

Marana, M.H., Chettri, J.K., Salten, M.B., Bach-Olesen, N.E., Kania, P.W., Dalsgaard, I. & Buchmann, K. (2020) Primary immunization using low antigen dosages and immunological tolerance in rainbow trout. *Fish Shellfish Immunol,* **105,** 16-23.

Olsen, M.M., Kania, P.W., Heinecke, R.D., Skjoedt, K., Rasmussen, K.J. & Buchmann, K. (2011) Cellular and humoral factors involved in the response of rainbow trout gills to *Ichthyophthirius multifiliis* infections: Molecular and immunohistochemical studies. *Fish & Shellfish Immunology,* **30,** 859-869.

Purcell, M.K., Kurath, G., Garver, K.A., Herwig, R.P. & Winton, J.R. (2004) Quantitative expression profiling of immune response genes in rainbow trout following infectious haematopoietic necrosis virus (IHNV) infection or DNA vaccination. *Fish Shellfish Immunol,* **17,** 447-462.

Raida, M.K. & Buchmann, K. (2007) Temperature-dependent expression of immune-relevant genes in rainbow trout following *Yersinia ruckeri* vaccination. *Diseases of aquatic organisms,* **77,** 41-52.

Raida, M.K. & Buchmann, K. (2008a) Bath vaccination of rainbow trout (*Oncorhynchus mykiss* Walbaum) against *Yersinia ruckeri*: effects of temperature on protection and gene expression. *Vaccine,* **26,** 1050-1062.

Raida MK, Buchmann K. (2008b)Development of adaptive immunity in rainbow trout, Oncorhynchus mykiss (Walbaum) surviving an infection with Yersinia ruckeri. Fish & Shellfish Immunology. 2008;25(5):533-41. doi: https://doi.org/10.1016/j.fsi.2008.07.008

Raida, M.K. & Buchmann, K. (2009) Innate immune response in rainbow trout (*Oncorhynchus mykiss*) against primary and secondary infections with *Yersinia ruckeri* O1. *Dev Comp Immunol,* **33,** 35-45.

Skov, J., Chettri, J.K., Jaafar, R.M., Kania, P.W., Dalsgaard, I. & Buchmann, K. (2018) Effects of soluble immunostimulants on mucosal immune responses in rainbow trout immersion-vaccinated against Yersinia ruckeri. *Aquaculture,* **492,** 237-246.

Skov, J., Kania, P.W., Holten-Andersen, L., Fouz, B. & Buchmann, K. (2012) Immunomodulatory effects of dietary beta-1,3-glucan from Euglena gracilis in rainbow trout (Oncorhynchus mykiss) immersion vaccinated against Yersinia ruckeri. *Fish Shellfish Immunol,* **33,** 111-120.

Xueqin, J., Kania, P.W. & Buchmann, K. (2012) Comparative effects of four feed types on white spot disease susceptibility and skin immune parameters in rainbow trout, *Oncorhynchus mykiss* (Walbaum). *Journal of Fish Diseases,* **35,** 127-135.
