# Supplementary material for: Responses towards eyefluke (Diplostomum pseudospathaceum) in different genetic lineages of rainbow trout
Source: PLoS One. 2022 Oct 27;17(10):e0276895. doi: 10.1371/journal.pone.0276895 (PMC9612474; doi:10.1371/journal.pone.0276895)
Supplement: S1 Fig — A. Gene expression result. First part of cytokine genes. The gene expression assessed using Two Way ANOVA. Significant regulation defined as: fold change at least 2 and p<0.05. *: p<0.05. Source of variation (p<0.05) is indicated by letters in circles above; G indicates Genotype; E indicates Exposure; I indicates Interaction between Genotype and Exposure. The y-axis represent geometric means and error bars geometric standard deviations. B. Gene expression result. Second part of cytokine and transcription factor genes. The gene expression assessed using Two Way ANOVA. Significant regulation was defined as both fold change was at least 2 and p<0.05. Asterisks indicate p<0.05. Source of variation (p<0.05) is indicated by letters in circles above; G indicates Genotype; E indicates Exposure; I indicates Interaction between Genotype and Exposure. The y-axis represent geometric means and error bars geometric standard deviations. C. Gene expression result. Immune cell marker genes. The gene expression assessed using Two Way ANOVA. Significant regulation defined as: fold change at least 2 and p<0.05. *: p<0.05. Source of variation (p<0.05) is indicated by letters in circles above; G indicates Genotype; E indicates Exposure; I indicates Interaction between Genotype and Exposure. The y-axis represent geometric means and error bars geometric standard deviations. D. Gene expression result. One marker for immune cells and 7 innate factors. The gene expression assessed using Two Way ANOVA. Significant regulation defined as: fold change at least 2 and p<0.05. *: p<0.05. Source of variation (p<0.05) is indicated by letters in circles above; G indicates Genotype; E indicates Exposure; I indicates Interaction between Genotype and Exposure. The y-axis represent geometric means and error bars geometric standard deviations. E. Gene expression result of the re-exposure trial. Only the 24 genes significantly regulated are shown. The gene expression assessed using Two Way ANOVA. Significant [file pone.0276895.s004.pdf]

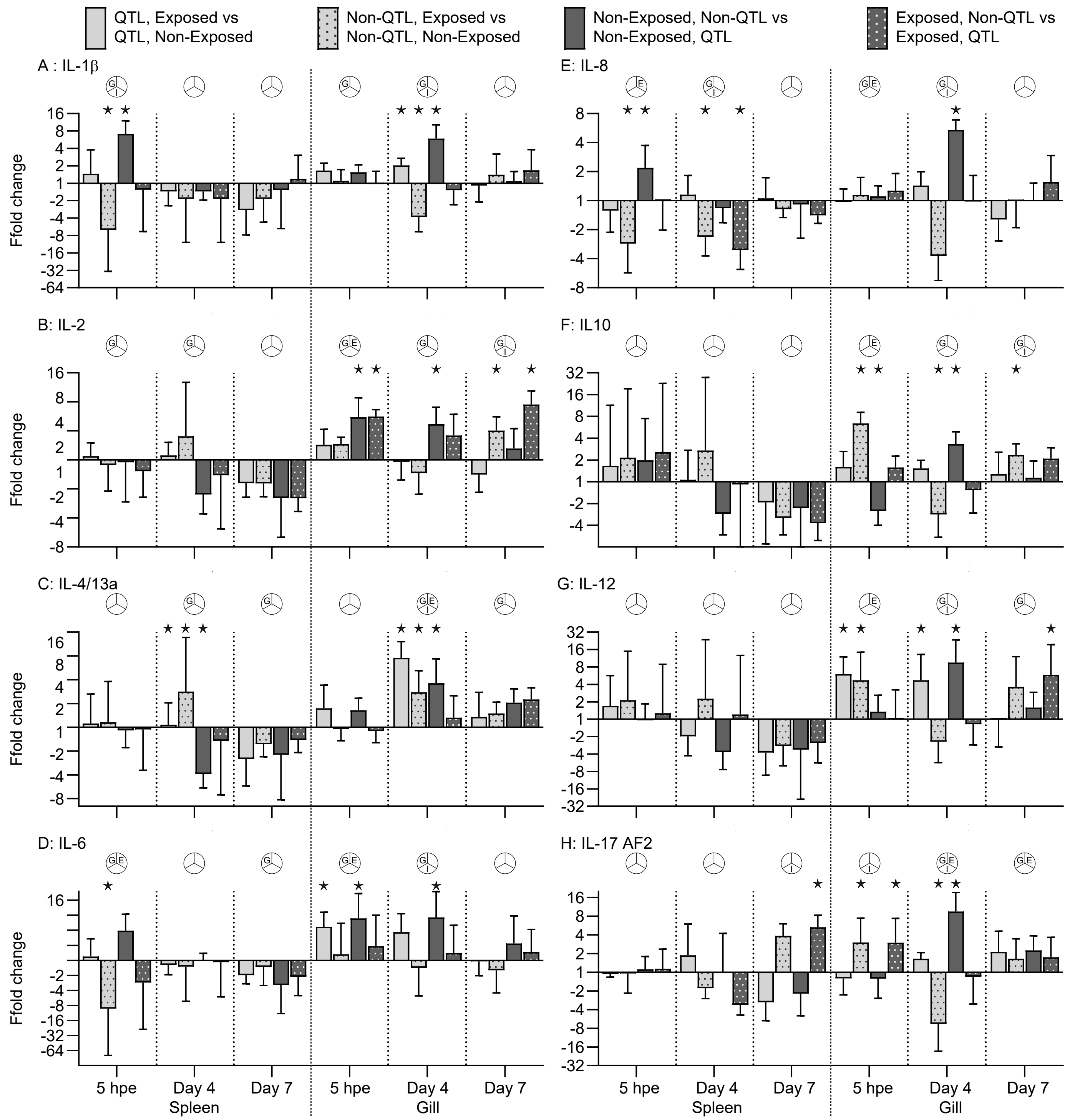

Fig S3A. Gene expression result. First part of the interleukins. The gene expression assessed using Two Way ANOVA. Significant regulation was defined as both fold change was at least 2 and p<0.05. Asterisks indicate p<0.05. Source of variation (p<0.05) is indicated by letters in circles above; G indicates Genotype; E indicates Exposure; I indicates Interaction between Genotype and Exposure. The y-axis's represent geometric means and error bars geometric standard deviations

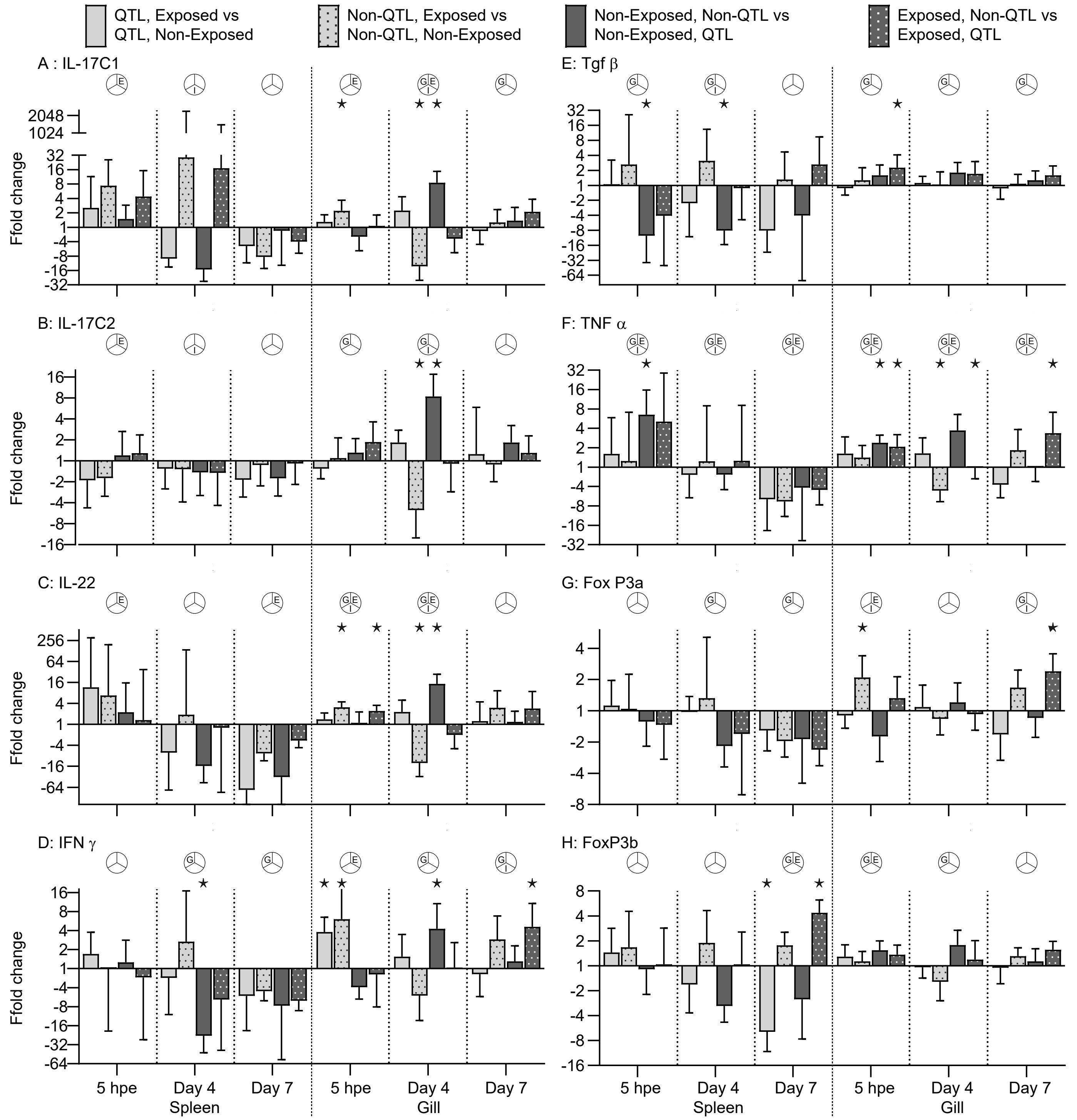

Fig S3B. Gene expression result. Second part of the interleukins, cytokines and transcription factors. The gene expression assessed using Two Way ANOVA. Significant regulation was defined as both fold change was at least 2 and $p<0.05$. Asterisks indicate $p<0.05$. Source of variation ($p<0.05$) is indicated by letters in circles above; G indicates Genotype; E indicates Exposure; I indicates Interaction between Genotype and Exposure. The y-axis's represent geometric means and error bars geometric standard deviations.

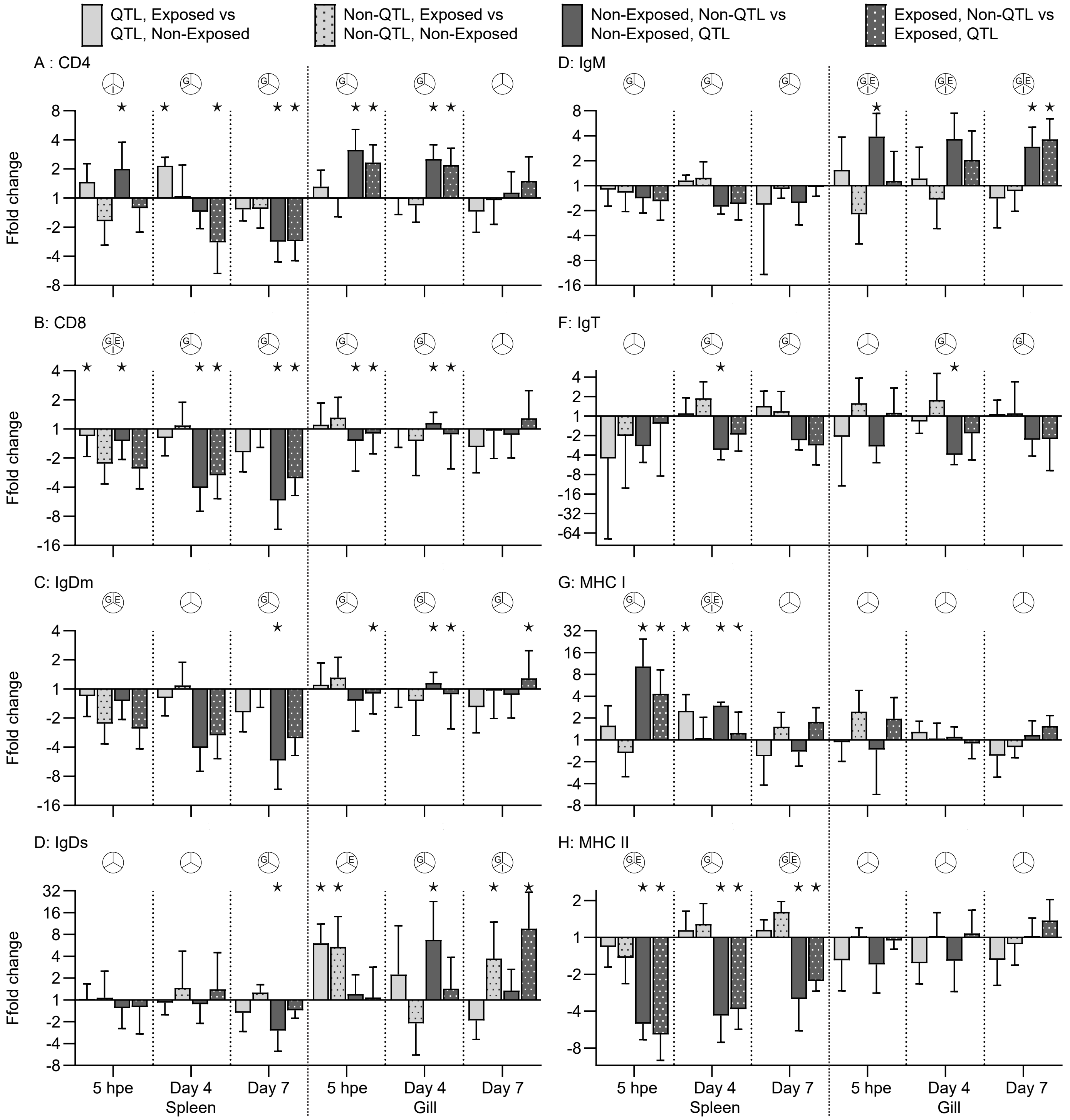

Fig S3C. Gene expression result. Markers for immune cells. The gene expression assessed using Two Way ANOVA. Significant regulation was defined as both fold change was at least 2 and p<0.05. Asterisks indicate p<0.05. Source of variation (p<0.05) is indicated by letters in circles above; G indicates Genotype; E indicates Exposure; I indicates Interaction between Genotype and Exposure. The y-axis's represent geometric means and error bars geometric standard deviations.

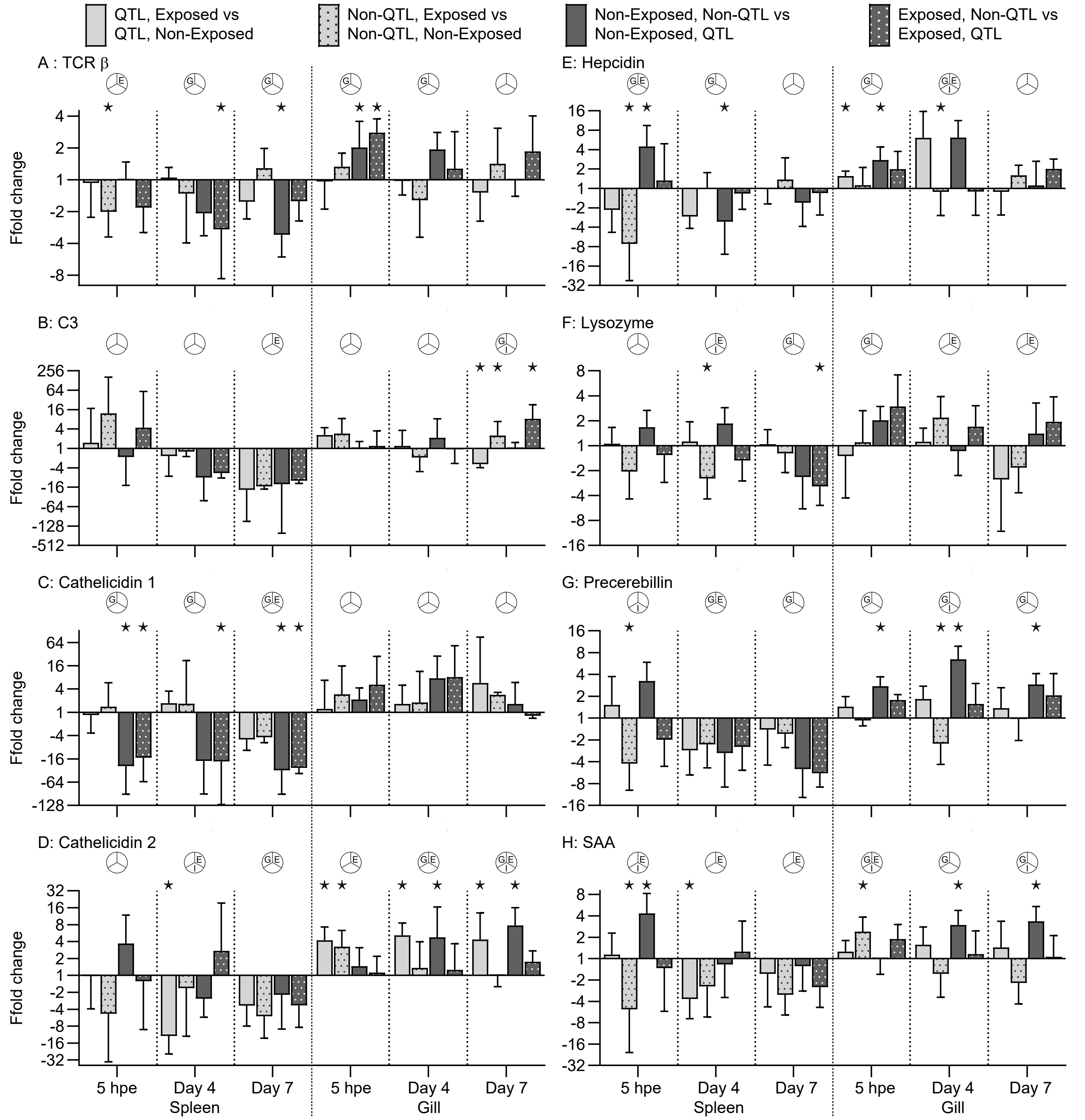

Fig S3D. Gene expression result. One marker for immune cells and 7 innate factors. The gene expression assessed using Two Way ANOVA. Significant regulation was defined as both fold change was at least 2 and p<0.05. Asterisks indicate p<0.05. Source of variation (p<0.05) is indicated by letters in circles above; G indicates Genotype; E indicates Exposure; I indicates Interaction between Genotype and Exposure. The y-axis's represent geometric means and error bars geometric standard deviations.

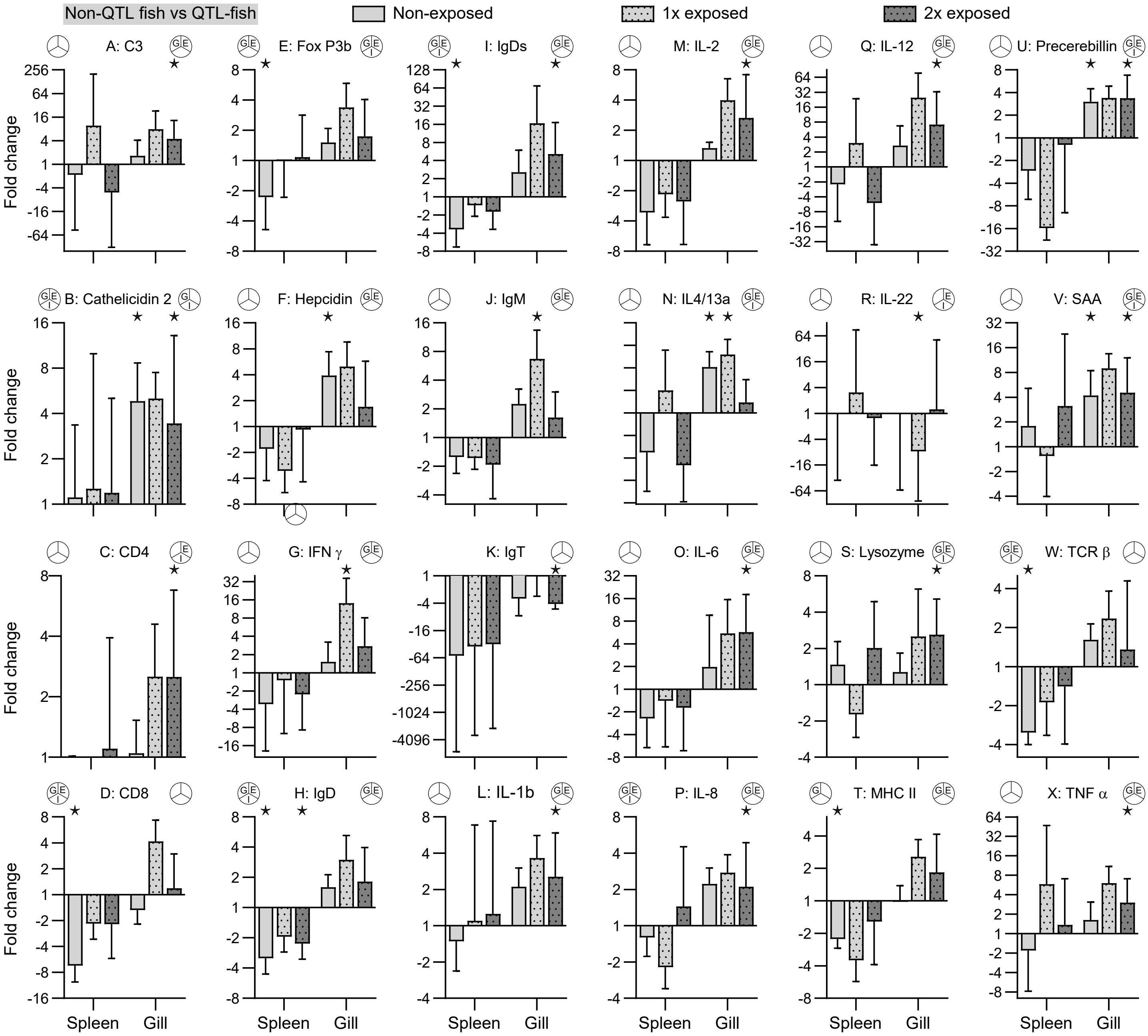

Fig S3E. Gene expression result of the re-exposure trial. Only the 24 genes exhibiting significance are shown. The gene expression assessed using Two Way ANOVA. Significant regulation was defined as both fold change was at least 2 and p<0.05. Asterisks indicate p<0.05. Source of variation (p<0.05) is indicated by letters in circles above; G indicates Genotype; E indicates Exposure; I indicates Interaction between Genotype and Exposure; results for spleen an gill are placed to the left and right, respectively. Please note the figure focus on the genotypes non-QTL fish versus QTL fish. The y-axis's represent geometric means and error bars geometric standard deviations.
